# Supplementary material for: Observed trends and projections of temperature and precipitation in the Olifants River Catchment in South Africa
Source: PLoS One. 2022 Aug 9;17(8):e0271974. doi: 10.1371/journal.pone.0271974 (PMC9362909; doi:10.1371/journal.pone.0271974)

Manuscript Click here to access/download;Manuscript;Manuscript_PlosOne.docx

Trends and projections of surface temperature and rainfall over the Olifants River catchment

Abiodun Morakinyo Adeola[1,2,3¶*], Andries Kruger[1,3¶], Thabo Elias Makgoale[1&], Joel Ondego. Botai[1,3,4&]

[1]South African Weather Service, Private Bag X097, Pretoria 0001, South Africa.

abiodun.adeola@weathersa.co.za; andries.kruger@weathersa.co.za; thabo.makgoale@weathersa.co.za; joel.botai@weathersa.co.za;

[2]School for Health Systems and Public Health, University of Pretoria, Pretoria, South Africa.

[3]Department of Geography, Geoinformatics and Meteorology, University of Pretoria, Pretoria, South Africa.

[4]School of Agricultural, Earth and Environmental Sciences, University of KwaZulu- Natal, Westville Campus, Durban, South Africa

* Corresponding author. abiodun.adeola@weathersa.co.za

Abstract

Within the climate change matrix, water resources are at the epicenter of projected climate change impacts. Certainly, the southern Africa climate is changing, consequently, localized studies are needed to determine the magnitude of anticipated changes for effective adaptation. This study examines trends in temperature and rainfall using historical observation data over the Olifants River Catchment for the period 1976–2019; as well as an ensemble of eight regional climate model (RCA4) simulations of the CORDEX Africa initiative. Future climate change projections under the "business as usual" climate scenario RCP 8.5 for two time periods of 2036-2065 (near future) and 2066-2095 (far future) were analysed. Trends and the statistical significance thereof, of the annual and seasonal series were calculated using the modified Mann-Kendall test. The results indicate that the catchment has experienced increases in the frequency of warm temperature and decline in rainfall, although no significant change in the distribution between future and historical rainfall was discernible. Also, surface temperature is expected to increase significantly, continuing from already significant historical trends in the mean temperature. Seasonally, the frequency of warming in minimum temperature is stronger in the summer season of December, January, February (DJF) and spring season of September, October, November (SON) compared with winter season of June, July, August (JJA) and autumn season of March, April, May (MAM). The results of this study provide an understanding of the characteristics of changing temperature and rainfall, in the context of the present anthropogenic global warming, and are relevant for decision-making processes, particularly for the sustainability of the Olifants River Catchment and its resources.

Keywords: Temperature, rainfall, trends, projections, seasonal, Olifants River Catchment, South Africa

Introduction

Climate change in Sub-Saharan Africa (SSA) is poised to affect people more than any other region because of its high exposure and lack of adaptation capability [1]. It is estimated that climate change will increase existing food insecurity, health challenges, poverty, and development challenges in the [2]. SSA is one of the areas where adaptation planning has been hindered by the complexity and relative uncertainty surrounding climate change impacts [3]. While uncertainties surround the understanding of future climate impacts in most regions of the globe, the SSA has among the lowest levels of confidence due to a lower resolution of available climate models and fewer research conducted [1]. Therefore, increasing the level of confidence in the projections for this region, or at least quantifying the scale of the uncertainty will contributes greatly towards providing the certainty required for effective adaptation planning.

Southern Africa is particularly indicated to be susceptible to climatic changes due to its high levels of economic inequality and heavy reliance on rain-fed agriculture [4]. Extreme weather events are widely projected to become more frequent, intense and severe as the global climate continues to warm due to anthropogenic greenhouse gas emissions [5]. South Africa, like other countries in the southern region, have recently witnessed increases in the frequency and intensity of extreme weather conditions such as the tropical cyclones Idai, Kenneth and the recent tropical cyclone Eloise, the fourth major cyclone to hit Southern Africa since 2017. These weather systems left behind them huge traces of economic destruction including losses of life and properties [6].

Climate change is anticipated to worsen existing challenges in various climate sensitive sectors, increasing their vulnerability as well as the human systems that depend on them [7-8]. Consequently, many studies have been conducted on analysing or generating possible near to far term future projections of likely changes in climatic variables [9]. Therefore, the extent to which climate and extreme weather events will change at local level, as the global climate warms from current levels to near, medium and far futures, need to be understood to allow for better preparedness and informed policy formulation. Globally, the overall

impacts of climate change on freshwater resources are projected to be negative. Furthermore, while the link between increased temperatures and changes in rainfall has been modelled in detail, the same is not true for the effect on specific river catchments and the recharge of underground waters.

Challenges posed by the changes in the frequency, duration and intensity of climatic variables coupled with increasing population and demand for social and economic equity are inevitable and ought to be well understood. Hence, the development of effective resource management, planning and adaptation strategies can be achieved through better understanding of the dynamics of current variability and future climate change [10]. In order to achieve enhanced capacity and adaptation strategies to respond to these inherent dynamics, climate change studies are needed at local scale (fine resolution) [11-13]. These strengthens resilience to current climate challenges, while building capacity to adapt to future climate change [10].

~~Studies have found that in order to make the most effective decisions, policy planning, and the proper implementation of adaptation projects and climate services, better short-term and mid-term climate projections are needed~~. [14]. Climate change projections have so far been conducted at the regional level in Southern Africa and SSA more specifically, using general circulation models (GCMs) [15-16]. Regional studies have shown that GCMs are able in reproducing temperature trends realistically. However, they tend to overestimate precipitation over all seasons for Southern Africa [17-18]. Furthermore, GCMs are relatively good to produce projections over a wide area, however, to determine context-specific impacts and adaptation measures require a deeper knowledge of the local conditions [19]. Consequently, studies have begun using regional downscaling techniques as a means of achieving greater levels of detail and improving projection accuracy [20-22]. For instance, [17] reported with a high level of confidence that Regional Climate Models (RCMs) can significantly contribute to climate projections especially in areas with highly heterogeneous topography, and when dealing with extreme or small-scale climatic processes. In addition, [23-24] showed that an ensemble of ten RCMs effectively simulates precipitation distribution patterns over Southern and Eastern Africa respectively.

Further, [22] indicated that the downscaled Met Office's global-scale Unified Model for the Africa-wide domain enabled the inclusion of additional details, such as higher resolutions and convection parametrizations. In their study they observed that the addition of convection enhanced rainfall simulation during the June-August period. Typical GCM-based models inherit biases within their boundary conditions; however, [18] demonstrated that the use of local climate forcings and RCM formulation reduces these biases in the African region. Studies have shown that RCMs can provide a better representation of annual cycles in Southern Africa and provide a greater level of detail in projections [12,18,25].

This study focusses on investigating the trends in historical maximum, minimum temperatures and rainfall, the estimation of plausible future climate changes, as well as the magnitude of future occurrences in the variables to inform adaptation initiatives over the Olifants River Catchment. This analysis provides a basis for future impact analysis and the development of effective adaptation strategies for various climate sensitive sectors within the catchment.

Materials and Methods

Study area

The Olifants River Catchment falls within the Limpopo River Basin, which is part of an international drainage basin that stretches across South Africa, Mozambique, Zimbabwe and Botswana (Fig 1). It has a total area of about 54,475 km^2 with about 3.2 million population, of which two-thirds are living in the rural communities [26]. In fact, the Olifants River contributes nearly 40% of the water that flows in the Limpopo River, making it an important river in the larger drainage system. The Olifants River flows for 560 km from Gauteng, through the Mpumalanga and Limpopo Provinces of South Africa passing through Mozambique and into the Indian Ocean. Major economic activities within the catchment include mining, industry and agriculture, which are largely dependent on water resources, and make up for about 6% of the gross domestic product of South Africa [27]. The catchment receives its greatest rainfall between October and April, with an average annual rainfall of 500 to 800 mm in the larger parts of the catchment

but, however, reaches 1000 mm along the escarpment [28]. With elevations ranging from 200m to about 2300m above sea level having a cool winter and year-round temperatures ranging from 0 °C to 35 °C.

Insert Figure 1 here:

Fig 1: Map showing (A) location of Olifants River Catchment in South Africa context, (B) location of temperature stations and delineation of numbered homogenous rainfall regions used for analysis

Data set

The climate data was obtained from the South African Weather Service (SAWS) climate database. There are about four observing stations within the proximity of the catchment which have sufficiently long data records. The data were quality controlled such that any station with 20% or more missing values were excluded from the analysis. Consequently, only two stations with data for the period of 1976 – 2019 were available for analysis over the study area. The climate data used include the rainfall, minimum temperature (Tmin) and maximum temperature (Tmax). Locations of the stations within the study area and the completeness of their records are shown in Fig 1 and Table 1.

Table 1: Summary of Temperature Stations with location and elevation

Name	Latitude	Longitude	Elevation (m)	Start Date
Hoedspruit	-24.35	31.05	520	1976
Oudestad	-25.18	29.34	955	1976

In addition, the South African Weather Service (SAWS) district rainfall dataset was analysed. The dataset is a delineation of areas with homogeneous rainfall, with South Africa divided into a total of 94 rainfall districts by SAWS [29], presented in Fig 1A. The delineation of these districts is mainly done according to the annual march of maximum rainfall, and the boundaries between the winter, whole year and summer rainfall regions. A district rainfall total is calculated as the average of the measured values from available or operational rainfall stations in the district on a monthly basis [30]. For this study, the relevant rainfall districts can be considered to be topographically less complex than most of the rainfall districts in South Africa, which makes the reliability of the monthly statistics relatively reliable. Fig 1B

presents the rainfall districts overlaid on the catchment areas. Consequently, rainfall totals over two rainfall districts of (34 and 63) corresponding to the temperature stations were used.

Additionally, an ensemble of eight individual Global Climate Models (GCMs) that participated in the Fifth Phase of Coupled Model Inter-comparison Project (CMIP5) (Table 2) dynamically downscaled to a finer spatial resolution (0.44° x 0.44°) by the Rossby Centre regional (RCA4) model was used for the future projections. The RCA4 is a coupled ocean-atmosphere Regional Climate Model (RCM) based on the Numerical Weather Prediction (NWP) model HARLAM [31]. The RCA4 simulated projections formed part of the Coordinated Regional Climate Downscaling Experiment (CORDEX) [32].

Table 2: List of the Global Circulation Models (GCMs) used in the study

Model name	Country	Resolution	Literature
**CanESM2m**	Canada	2.8° x 2.8°	[33]
**CNRM-CM5**	France	1.4° x 1.4°	[34]
**CSIRO-Mk3**	Australia	1.9° x 1.9°	[35]
**IPSL-CM5A-MR**	France	1.9° x 3.8°	[36]
**MICRO5**	Japan	1.4° x 1.4°	[37]
**MPI-ESM-LR**	Germany	1.9° x 1.9°	[38]
**NorESMI-M**	Norway	1.9° x 2.5°	[39]
**GFDL-ESM2M**	USA	2.0° x 2.5°	[40]

Analysis

Statistical and trend analysis

The daily temperature and rainfall data were aggregated to monthly, seasonal and annual data. A boxplot analysis was used to examine the general statistical characteristics of the data. The annual and seasonal time-series was analyzed for trends using the Mann-Kendall (MK) test statistic S presented in Equation 1 [41]. The MK is a non-parametric test with the advantages of making no assumptions about the distribution of the underlying data and being relatively insensitive to outliers [41].

$$S = \sum_{k=1}^{n-1} \sum_{j=k+1}^{n} sgn\left(x_j - x_k\right) \quad (1)$$

$$sgn\left(x_j - x_k\right) = \begin{cases} 1\ if\ x_j - x_k > 0 \\ 0\ if\ x_j - x_k = 0 \\ -1\ if\ x_j - x_k < 0 \end{cases}$$

where, x_j and x_k are the annual or seasonal values in years j and k respectively.

An upward or a downward trend is indicated when S is positive or negative respectively. In order to determine whether a trend is statistically significant, the Z value is used. Evidently, a positive Z value indicates an upward trend and a negative Z value indicates a downward trend. To test for either an upward or downward monotone trend (a two-tailed test), the 95% level of confidence was applied. Hence, for statistical significance, the 95% level of confidence is such that if the probability estimate is equal to or less than 0.05, the trend is deemed to be significant.

The change per unit time (annual or seasonal) within the time series was estimated using the Sen's nonparametric approach with an assumption that the trend is linear [42]. The magnitude of the trend is predicted by the Sen's estimator. The slope is computed using equation 2. A positive or negative value of Q_i indicates an increasing or decreasing rate, respectively.

$$Q_i = \frac{(x_j - x_k)}{j - k}\ for\ i = 1,2,3\ \ldots \quad (2)$$

Where, x_j and x_k are the annual or seasonal values in years j and k respectively.

Model evaluation

The reliability of the individual model and ensembles to simulate observed annual and seasonal data was assessed using the Taylor diagram [43]. The diagram is a visual representation of how closely a pattern fits observation. The Taylor diagram is quantified in terms of the correlation (R), the centered root-

mean-square-error (*RMSE*) and the amplitude of the standard deviations (*Std*). Moreover, the uncertainty of the different multi-model ensembles is also compared. The three statistics are integrated by equation 3;

$$E' = {\sigma_o}^2 + {\sigma_s}^2 - 2\sigma_o\sigma_s\rho \qquad 3$$

where E' is the centered root-mean-square-error, σ_o is the standard deviation of observed values, σ_s is the standard deviation of simulated values and ρ is the correlation coefficient between the simulated and observed values.

For a given linear analysis of model, a model with low variance and low bias is considered as the ideal model and most suitable for projection while a model with low bias and high variance is considered as a model with overfitting; a model with high bias and low variance is typically an underfitting model. In addition, a model with high bias and high variance will assuredly probably give the greatest prediction error.

Climate change projections

Daily model simulated values of total rainfall, ~~and~~ maximum and minimum temperature averages are used to generate projections of annual change, as well as projections of the 3-month seasonal change. Future projections of rainfall, maximum and minimum temperature are presented for the three 30-year periods extending from 2036 – 2065 (near future) and 2066 – 2095 (far future) under the RCP 8.5 scenario. Projected changes are expressed relative to the historical 30-year period of 1976 to 2005. The seasons considered are December-January-February (DJF), March-April-May (MAM), June-July-August (JJA) and September-October-November (SON). Additionally, the Probability Distribution Function was used to estimate the occurrence (number of possible events) of both the historical and projected Tmax, Tmin and rainfall generated over the entire catchment.

Results

Historical variations

Fig 2 shows box plots of Tmax, Tmin and rainfall with the quantile values for minimum, first quartile, median, third quartile, and maximum for the two weather stations and corresponding homogenous rainfall districts for the period under investigation. At a monthly average temperature (Tmean) of 25.9 and 24.5 °C January is the hottest month of the year over the northern part (Hoedspruit station) and southern part (Oudestad station) of the catchment respectively. At 17.3 and 13.2 °C on average, July is the coldest month of the year over the northern part and southern part of the catchment respectively. The southern part of the catchment is wetter with larger rainfall received in January. Between the driest and wettest months, the difference in rainfall is 80.0 mm and 103.6 mm over Hoedspruit and Oudestad stations respectively (Table 3)

Insert Figure 2 here:

Fig 2: Boxplot of minimum temperatures (A top; B top) and maximum temperature (A bottom; B bottom) over Hoedspruit (A) and Oudestad (B) stations and rainfall (C) top District 34 and bottom District 63

Table 3: Mean monthly values of Maximum temperature (Tmax), Minimum temperature (Tmin), Average Temperature (Tmean) and Total Rainfall over the period of study (1976-2019)

Month	Hoedspruit/District 34				Oudestad/District 63			
	Tmax (°C)	Tmin (°C)	TMean (°C)	Rainfall (mm)	Tmax (°C)	Tmin (°C)	TMean (°C)	Rainfall (mm)
Jan	31.4	20.4	25.9	83.0	31.1	17.9	24.5	106.3
Feb	31.3	20.3	25.8	79.1	31.2	17.5	24.3	79.1
Mar	30.6	19.3	25.0	52.8	30.0	16.0	23.0	71.7
Apr	28.6	16.5	22.6	26.3	27.7	12.1	19.9	32.3
May	27.2	12.8	20.0	8.4	25.4	7.7	16.5	11.2
Jun	25.3	10.0	17.7	2.9	22.7	4.3	13.5	4.3
Jul	24.7	9.8	17.3	4.8	22.6	3.8	13.2	2.7
Aug	26.6	11.8	19.2	4.9	25.3	6.4	15.8	6.3
Sep	28.6	14.5	21.6	10.0	28.7	10.9	19.8	17.1
Oct	29.3	16.6	22.9	29.3	29.8	14.4	22.1	64.3
Nov	30.4	18.4	24.4	64.0	30.0	16.1	23.1	105.0
Dec	31.4	19.7	25.6	81.1	30.8	17.5	24.1	119.7

Trends

The descriptive statistics and historical trends of annual and seasonal rainfall, Tmax and Tmin for the two climate stations and corresponding homogenous rainfall districts with long term data are presented on seasonal and annual bases from 1976 – 2019 in Table 4. In the northern part of the Catchment at Hoedspruit station, Tmean ranged between 27.3 to 30.2 °C with an average value of 28.8 ± 0.7 °C for the period 1976 to 2019. The Mann-Kendall trend analysis showed that test Z was +0.201 and Sen's slope estimator was +0.018, which indicated that mean annual Tmax significantly increased at the rate of 0.018 °C per year at 95% confidence level for the period under study. On the other hand, the mean annual Tmin varied between 14.3 to 17.8 °C with a mean value of 15.9 ± 0.7 °C (Table 4). The trend analysis of mean annual Tmin showed that it decreased with a Z = -0.086 at the rate of 0.008 °C per year for the period of study. However, unlike the maximum temperature, the rate of decrease of mean annual minimum temperature was not significant at the 95% confidence level. Seasonally, Tmax shows an increasing trend across all seasons. However, the increasing trend is only statistically significant for Tmax in winter and spring. The results indicate a decreasing trend in Tmin for all the seasons except for winter, however, only summer Tmin is statistically significant. Overall, Tmean have increased by 0.4 and 0.2 °C respectively in the northern part. Although, the rainfall shows a decreasing trend, the trends are not statistically significant.

In the southern part of the Catchment at the Oudestad climate station, the mean annual Tmax ranged between 26.1 to 31.3 °C with an average value of 27.9 ± 1.1 °C for the period 1976 to 2019. The Mann-Kendall trend analysis showed that test Z was +0.53 and Sen's slope estimator was +0.055, which indicated that mean annual Tmax significantly increased at the rate of 0.055 °C per year at the 95% confidence level for the period under study. On the other hand, the mean annual Tmin varied between 9.5 to 13.9 °C with a mean value of 11.9 ± 0.7 °C (Table 4). The trend analysis of mean annual Tmin showed that it increased with a Z = 0.18 at the rate of 0.018 °C per year for the period of study. However, the rate of increase of mean annual Tmin was not significant at the 95% confidence level. Seasonally, both Tmin and Tmax show increasing trends across all seasons. The increasing trends are statistically significant for Tmax for all the

four seasons but statistically not significant for Tmin in all the four seasons at the 95% confidence level. In general, mean daily Tmax and Tmin have increased by 0.42 and 0.27 °C respectively in the southern part of the Catchment.

The rainfall does not show a clear signal for increasing or decreasing trend. A decreasing trend in total annual and seasonal rainfall in the northern (district 34) of the study area is indicated by the results. However, the trends are not statistically significant at the 95% confidence level. The mean total annual rainfall varied from 176.9 to 988.1 mm with a mean of 446.6 mm±160.4. In the southern (district 63) parts, the mean total annual rainfall varied from 385.6 to 852.7 mm with a mean of 620.0 mm±96.1 The Mann-Kendall trend analysis showed that test Z was +0.066 with a Sen's slope estimator of +0.007, which indicated that total annual rainfall has increased over the study period. In addition, the results indicate increasing trend in total seasonal rainfall in summer. However, the trends are not significant at the 95% confidence level.

Table 4: Descriptive statistics and trend analysis of maximum, minimum temperature (°C) and rainfall (mm)

Location	Statistics	Average maximum temperature (°C)					Average minimum temperature (°C)					Rainfall (mm)				
		**Summer**	**Autumn**	**Winter**	**Spring**	**Annual**	**Summer**	**Autumn**	**Winter**	**Spring**	**Annual**	**Summer**	**Autumn**	**Winter**	**Spring**	**Annual**
Hoedspruit (DS34)	Maximum	34.8	34.6	29.0	33.0	30.2	23.0	21.2	15.4	20.35	17.8	422.5	175.7	82.8	167.9	988.1
	Minimum	28.1	24.6	22.7	25.5	27.3	14.7	10.1	6.5	12.0	14.3	2.8	0.0	0.0	0.0	176.9
	Mean	31.4	28.8	25.6	29.4	28.8	20.2	16.3	10.5	15.5	15.9	81.1	29.2	4.2	34.4	446.6
	SD	1.4	2.1	1.3	1.5	0.7	1.1	2.9	1.8	1.9	0.7	61.62	32.0	9.7	34.4	160.4
	CV	4.6	7.3	5.1	5.1	2.4	5.6	18.1	16.9	12.09	4.7	76.02	109.5	230.4	100.0	35.9
	Z Test	+0.069	+0.051	+0.318	+0.242	+0.201	-0.21	-0.139	+0.076	-0.024	-0.086	-0.165	-0.010	-0.11	-0.162	-0.171
	Sen's slope	+0.033	+0.024	+0.149	+0.115	+0.018	-0.104	-0.066	+0.036	-0.012	-0.008	-0.084	-0.051	-0.057	-0.082	-0.017
	Trend	Increase	Increase	**Increase**	**Increase**	**Increase**	Decrease	Decrease	Increase	Decrease	Decrease	Decrease	Decrease	Decrease	Decrease	Decrease
	(p-value) Significance	0.49 NS	0.61 NS	**0.001 S**	**0.02 S**	**0.04 S**	**0.03 S**	0.16 NS	0.45 NS	0.81 NS	0.39 NS	0.09 NS	0.31 NS	0.26 NS	0.11 NS	0.087 NS
Oudestad (DS63)	Maximum	36.4	33.8	29.7	34.9	31.3	19.9	18.8	8.8	18.1	13.9	224.6	181.1	29.2	213.3	852.7
	Minimum	25.80	22.7	19.95	25.1	26.1	14.8	4.7	0.8	7.2	9.5	24.4	0.1	0	0.0	385.6
	Mean	31.0	27.7	23.43	29.46	27.9	17.6	12.1	4.7	13.8	11.9	101.7	38.4	4.4	62.13	620.0
	SD	1.6	2.4	1.8	1.8	1.1	0.9	3.6	1.6	2.5	0.7	39.9	34.83	6.9	48.2	96.1
	CV	5.2	8.7	7.9	6.1	4.0	5.3	30.2	34.7	17.9	5.7	39.3	90.72	156.6	77.5	15.5
	Z Test	+0.46	+0.27	+0.596	+0.645	+0.53	+0.062	+0.071	+0.171	+0.032	+0.18	+0.067	+0.026	-0.12	-0.038	+0.066
	Sen's slope	+0.24	+0.14	+0.303	+0.328	+0.055	+0.032	+0.036	+0.087	+0.017	+0.018	+0.034	+0.013	-0.062	-0.019	+0.007
	Trend	**Increase**	**Increase**	**Increase**	**Increase**	**Increase**	Increase	Increase	Increase	Increase	Increase	Increase	Increase	Decrease	Decrease	Increase
	(p-value) Significance	**0.003 S**	**0.01 S**	**0.003 S**	**0.001 S**	**0.001 S**	0.53 NS	0.48 NS	0.09 NS	0.75 NS	0.07 NS	0.50 NS	0.79 NS	0.22 NS	0.70 NS	0.511 NS

Significance: S- Significant, NS- Not Significant

Model evaluation

The results of the model performance to simulate historical temperatures and rainfall are shown in Figs 3 and 4 for Hoedspruit and Oudestad stations respectively. Statistics for eight models were calculated with each model and ensemble given a colour code. The location of each colour on the plot indicates how closely the model's simulated variables (rainfall and temperatures) matches the observed pattern. The results indicate a high correlation between the observed and model simulated annual Tmax while each model tends to weakly compare to the observations seasonally. On the other hand, high correlation is noticed between the observed and model simulated annual Tmin for autumn and spring. The ensemble mean of the member models produces a higher correlation between the observed and model simulated Tmax and Tmin at all time scales; however, there still exist a weak correlation between the ensembled model and the observed rainfall.

Insert Figure 3 here:

Fig 3: Performance and comparison of each model and ensemble against the actual observations of (top) maximum and (bottom) minimum temperatures at the Hoedspruit station.

Insert Figure 4 here:

Fig 4: Performance and comparison of each model and ensemble against the actual observations of (top) maximum and (bottom) minimum temperatures at the Oudestad station.

Climate change projections for Olifants River Catchment

Projected changes in the annual values of total rainfall, mean Tmax and Tmin over the Olifants River Catchment for the periods 2036 – 2065 (near future) and 2066 – 2095 (far future) under the RCP8.5 scenarios, relative to reference period 1976 – 2005 is presented in Fig 5. Generally, the projected changes in temperature and rainfall vary substantially across the catchment. These variations can be attributed to the complexity of the landscape and the climate fluctuations as shown in the historical trend analysis. At mid-century 2050 (2036-2065), Tmax is projected to be warmer by about 2.2 °C in the northern part and by 2.5 °C in the southern part of the catchment, compared to the baseline period of 1976-2005. On the other hand, Tmin is projected to increase by about 1.6 °C in the northern and central parts, and by about 2.0 °C in the

southwest. The warming trend is projected to further increase into the far future towards the end of the century (mid 2080) under the "Business as Usual" scenario. Tmax is projected to increase by about 5 °C in the southern part and 3.8 °C in the northern part whereas Tmin is estimated to increase by about 4.3 °C in the southwestern part and by about 3.6 °C in the northern and central parts, relative to the baseline period of 1976-2005.

Insert Figure 5 here:

Fig 5. Projected changes in annual (A) mean maximum, (B) minimum temperature and (C) total rainfall, for 2036 – 2065 (2nd column) and 2066 – 2095 (3rd column) periods under scenarios of the RCP8.5 relative to baseline period 1976-2005 (1st column) over the Olifants River Catchment.

It is important to note that there is high uncertainty in the direction and amount of change in mean annual precipitation across the ensemble models. The projection suggests a decrease in total annual rainfall by about 5-30%, especially over the eastern to central parts of the catchment by mid-2050. Areas with historically less rainfall is projected to have marginal increases in rainfall. The southern region of the catchment is projected to receive about a 20mm (5%) increase in rainfall. The results indicate a further decrease in rainfall (about 10-40%) over a large portion of the catchment going into the far future. The central interior part of the catchment is projected to experience a significant decrease in annual rainfall up to about 25%, with increased drying over time. Despite predictions of general drying conditions over most of the catchment, slight to moderate rainfall increases are projected over the south-western parts of Olifants River Catchment in spring and summer, although statistically not significant.

The results of the occurrence of both the historical and projected Tmax, Tmin and rainfall generated using the probability distribution function over the entire catchment are shown in Figs 6-10. The Figs illustrate the averages of the variables as well as the lower and upper extreme boundaries of the projected climate. As shown in Fig 6, the number of days (events) with annual maximum temperature > 32 °C is projected to increase by 2036-2065 (Fig 6E).

Insert Figure 6 here:

Fig 6: Probability distribution estimation of daily seasonal A) Summer, B) Autumn, C) Winter, D) Spring and E) annual maximum temperatures Over Olifants Catchment (1976 – 2005 vs. 2036 – 2065)

Seasonally, mean Tmax is expected to increase above 31 °C in summer, 29 °C in autumn, 26 °C in winter and 31 °C in spring by the mid-2050s (Fig 6A, B, C and D respectively). These maximum temperature values are projected to further increase in the far future reaching above 33 °C in summer, 31 °C in autumn, 26 °C in winter and 32 °C in spring (Fig 7A, B, C and D respectively) with increasing number of occurrences.

Insert Figure 7 here:

Fig 7: Probability distribution estimation of daily seasonal A) Summer, B) Autumn, C) Winter, D) Spring and E) annual maximum temperatures Over Olifants Catchment (1976 – 2005 vs. 2066 – 2095)

On the other hand, Tmin > 17 °C is projected to increase over the catchment (Fig 8E) in the mid-2050s. Seasonally, the number of events of Tmin > 18 °C is projected to increase in the summer months. In general, all seasons are projected to be experience higher Tmin under the RCP8.5 scenario by mid-2050s (Fig 8A, B, C and D respectively).

Insert Figure 8 here:

Fig 8: Probability distribution estimation of daily seasonal A) Summer, B) Autumn, C) Winter, D) Spring and E) annual minimum temperatures Over Olifants Catchment (1976 – 2005 vs. 2036 – 2065)

By mid-2080s minimum temperature values are further projected to increase above 19 °C with increased days of occurrence in summer (Fig 9A). Average Tmin values are projected to increase above 17 °C, 11 °C and 16 °C in autumn, winter and spring respectively (Fig 9B, C and D).

Insert Figure 9 here:

Fig 9: Probability distribution estimation of daily seasonal A) Summer, B) Autumn, C) Winter, D) Spring and E) annual minimum temperatures Over Olifants Catchment (1976 – 2005 vs. 2066 – 2095)

Fig 10 presents the histograms of annual and seasonal monthly total rainfall for the historical period (1976 – 2005) and projected (2036 – 2065) indicating the expected changes in the annual and seasonal rainfall distributions over the Olifants catchment, as predicted by the CORDEX multi-model mean. The Fig shows that annual total rainfall is projected to reduce in amount and in the number of rainfall events (Fig 10E). The declining trend in total rainfall is the same across the season except for a projected increase in the events of rainfall amount > 450 mm in summer and > 300 mm in spring.

Insert Figure 10 here:

Fig 10: Probability distribution estimation of seasonal and annual total precipitation over the Olifants catchment (1976 – 2005 vs. 2026 - 2055) with relevant gamma distributions superimposed

Discussion

The historical analysis indicates increasing trends in temperature and generally decreasing trend in rainfall. It is anticipated that future temperature will increase, and rainfall decrease under the business-as-usual RCP8.5 scenario over the Olifants River Catchment. The findings of this study as related to temperature agrees with the findings of [44] who reported significant increasing trends in warm temperatures over individual stations located in the northern sections of South Africa. In addition, the results of the decreasing trends in observed rainfall is consistent with the individual station analysis conducted by [30]. The study found that there are long-term increases in the southern interior and decreases in the far north-east, but no significant trends in annual and seasonal rainfall over most of the remainder of South Africa.

While there is no strong correlation between simulated and observed rainfall, climate models can still be used since they are not meant to predict specific weather events, but rather the trend in climatic change over time. However, we suggest that its usage should be guided by historical understanding. The results show a good correlation between simulated and observed temperatures. Hence, the utility of temperature projections is more reliable, particularly if used for estimating the rate of change in temperature rather than absolute values.

Given the historical increasing drying and warming trend in rainfall and temperatures over the catchment, climate change is expected to exacerbate the existing shortages in water supply with both direct and indirect impacts on other sectors. As temperatures rise and rainfall decreases, crop yield will be affected, and food security will be compromised. Particularly, farmers who rely heavily on rain-fed agriculture will be the most adversely affected by climate change. In addition, the projected climate changes in the catchment will have varying impacts on the various economic sectors. For example, human health would be impacted through increased incidence of malaria, cholera, and diarrhea [45]. Furthermore, the prediction points to a likely scenario of increasing temperatures together with more dry periods which in

turn points to an increased likelihood of severe droughts. With vast economic activities such as mining and agriculture in the catchment largely dependent on water resources (rainfall), the results of this research will be imperative to successfully develop an impact assessment and adaptation planning to safeguard the economic contribution of the region to the national GPD.

Conclusion

Climate induced threats are expected to increase in frequency and severity under climate change. In this study, trends in historical annual and seasonal temperatures as well as rainfall was examined, and future projections was investigated over the Olifants River Catchment in order to understand the vulnerability of the region to climate change. Daily maximum and minimum temperature from two ground weather stations and corresponding SAWS' district of homogenous monthly rainfall data was used. An ensemble of eight regional climate model simulations of the CORDEX Africa forced with CMIP5 models was used to make future climate change projections under the "business as usual" climate scenario RCP 8.5 for two time periods of 2036-2065 (near future) and 2066-2095 (far future). The ensemble of the RCMs is well able to depict the spatial distribution of temperatures and rainfall when compared with historical records. However, uncertainties are associated with rainfall direction and amount of change. The ensemble of RCMs outperformed individual models. The results indicate an increasing trend in historical and projected Tmax and Tmin between 1-5 °C, particularly in the southern part of the catchment and a decreasing trend in both historical and future rainfall of about 5-30% across the catchment. Major decline in the projected rainfall will occur over the central part of the catchment.

A general increasing trend in the number of days with warmer temperatures is projected over the catchment although with varied spatial distribution. In addition, decreasing trend in the number of annual rainfalls is projected for the remainder of the century, although the magnitude of trend varied over the catchment. Consequently, extreme events such as heatwaves and floods are expected to increase in occurrence in the future, particularly towards the end of the century. Such continued climatic changes are

a huge threat to general natural systems functioning coupled with other existing stressors such as increasing population growth, poverty, inequality and lack of strategic policy implementation.

The findings of these analyses are essential for the development of adaptation strategies for the various economic sectors functioning in the Olifants River Catchment. We suggest that government through the relevant structure should facilitate the provision and access to simplified information on anticipated changes in climatic variables and plausible impacts for varying climate sensitive sectors particularly agriculture and water. In addition, the provision of essential tools for decision making for present and future management and practices among direct users such as farmers are imperative for climate change adaptation purposes. Furthermore, given the results climate change is likely to be an additional challenge for achieving various sustainable development goals most desirables at local scales in South Africa. If no positive actions are taken, the goals of no poverty, zero hunger, good health and well-being, affordable and clean energy, and decent work and economic growth may remain a mirage.

Authorship contribution

Abiodun Adeola conceptualized the research, assisted by Andries Kruger and Thabo Makgoale analyzed the data. Abiodun Adeola and Andries Kruger wrote the initial manuscript. Joel Botai reviewed and edited the manuscript. All authors read and approved the final manuscript.

Conflicts of interest

The authors declare no conflicts of interest.

Acknowledgments

The authors present their warm thanks to South32 for inspiring the research.

Funding

Partial funding for the research was received from the South32 mining company.

Code and data availability

The data process is done using R Software that includes several packages for mapping NetCDF data. These can be made available upon request.

References

1. Niang, I., Ruppel, O.C., Abdrabo,M.A., Essel, A., Lennard, C., Padgham, J., Urquhart, P., 2014. In: Barros, V.R., Field, C.B., Dokken, D.J., Mastrandrea, M.D., Mach, K.J., Bilir, T.E., Chatterjee, M., Ebi, K.L., Estrada, Y.O., Genova, R.C., Girma, B., Kissel, E.S., Levy, A.N., MacCracken, S., Mastrandrea, P.R.,White, L.L. (Eds.), Africa. Climate Change 2014: Impacts, Adaptation, and Vulnerability. Part B: Regional Aspects. Contribution of Working Group II to the Fifth Assessment Report of the Intergovernmental Panel on Climate Change. Cambridge University Press, Cambridge, United Kingdom and New York, NY, USA.
2. Warnatzsch, E.A., Reay, D.S. (2019) Temperature and precipitation change in Malawi: Evaluation of CORDEX-Africa climate simulations for climate change impact assessments and adaptation planning. Science of the Total Environment 654, 378–392
3. Thornton, P.K., Jones, P.G., Owiyo, T., Kruska, R.L., Herrero, M., Kristjanson, P.M., Notenbaert, A.M.O., Bekele, N., Omolo, A., 2006. Mapping Climate Vulnerability and Poverty in Africa. ILRI, Nairobi, Kenya.
4. Strauss, J.A, Swanepoel, P.A, Laker MC & Smith, H.J (2021) Conservation agriculture in rainfed annual crop production in South Africa, South African Journal of Plant and Soil, DOI: 10.1080/02571862.2021.1891472.
5. Hegerl, G.C., F.W. Zwiers, V.V. Kharin, and P.A. Stott, 2004: Detectability of anthropogenic changes in temperature and precipitation extremes. J. Clim., 17, 3683–3700.
6. USAID. Southern Africa - Tropical Cyclones - Fact Sheet #12 FY2019. May 16, 2019. Available on https://www.usaid.gov/cyclone-idai/fy19/fs12
7. Thomas, D.S.G., Twyman, C., Osbahr, H. Hewitson B 2007. Adaptation to climate change and variability: farmer responses to intra-seasonal precipitation trends in South Africa. Climatic Change 83, 301–322. https://doi.org/10.1007/s10584-006-9205-4
8. Intergovernmental Panel on Climate Change (IPCC). 2014a: Climate Change 2014: Impacts, Adaptation, and Vulnerability. Available: http://www.ipcc.ch/report/ar5/wg2/
9. Kusangaya S, Warburton M.L., Garderen, E.A, Jewitt G.P.W. 2014 Impacts of climate change on water resources in southern Africa: A review, Physics and Chemistry of the Earth, Parts A/B/C, 67, 47-54, https://doi.org/10.1016/j.pce.2013.09.014.
10. Ziervogel G, Zermoglio F. Climate change scenarios and the development of adaptation strategies in Africa: challenges and opportunities. Clim Res 2009, 40:133–146.
11. Giorgi, F., Jones, C., Asrar, G.R., 2009. Addressing Climate Information Needs at the Regional Level: the CORDEX Framework. 58 pp. 175–183.
12. Nikulin, G., Jones, C., Giorgi, F., Asrar, G., Büchner, M., Cerezo-Mota, R., Bøssing Christensen, O., Déqué, M., Fernandez, J., Hänsler, A., Van Meijgaard, E., Samuelsson, P., Bamba Sylla, M. & Sushama, L., 2012. Precipitation climatology in an ensemble of CORDEX-Africa regional climate simulations. Am. Meteorol. Soc. J. Clim. 25, 6057–6078.
13. Zhang, Q., Körnich, H., Holmgren, K., 2013. How well do reanalyses represent the southern African precipitation? Clim. Dyn. 40, 951–962.

14. Nyamwanza, A.M., New, M.G., Fujisawa, M., Johnston, P., Hajat, A., 2017. Contributions of decadal climate information in agriculture and food systems in east and southern Africa. Clim. Chang. 143, 115–128.
15. Osborn, T., Wallace, C., Harris, I., Melvin, T., 2015. Climate projections - overview for specific global warming levels. Available Online: https://crudata.uea.ac.uk/~timo/climgen/national/web/Malawi/location.htm, Accessed date: 02 June 2020.
16. Serdeczny, O., Adams, S., Baarsch, F., Coumou, D., Robinson, A., Hare, W., Schaeffer, M., Perrette, M., Reinhardt, J., 2017. Climate change impacts in Sub-Saharan Africa: from physical changes to their social repercussions. Reg. Environ. Chang. 17, 1585–1600.
17. Flato, G., Marotzke, J., Abiodun, B., Braconnot, P., Chou, S.C., Collins, W., Cox, P., Driouech, F., Emori, S., Eyring, V., Forest, C., Gleckler, P., Guilyardi, E., Jakob, C., Kattsov, V., Reason, C., Rummukainen, M., 2013. In: Stocker, T.F., Qin, D., Plattner, G.-K., Tignor, M., Allen, S.K., Boschung, J., Nauels, A., Xia, Y., Bex, V., Midgley, P.M. (Eds.), Evaluation of Climate Models. Climate Change 2013: The Physical Science Basis. Contribution of Working Group I to the Fifth Assessment Report of the Intergovernmental Panel on Climate Change. Cambridge University Press, Cambridge, United Kingdom and New York, NY, USA.
18. Buontempo, C., Mathison, C., Jones, R.,William, K.,Wang, C., Mcsweeney, C., 2015. An ensemble climate projection for Africa. Clim. Dyn. 44, 2097–2118.
19. Kim, J.,Waliser, D.E., Mattmann, C.A., Goodale, C.E., Hart, A.F., Zimdars, P.A., Crichton, D.J., Jones, C., Nikulin, G., Hewitson, B., Jack, C., Lennard, C., A., F., 2014. Evaluation of the CORDEX-Africa multi-RCM hindcast: systematic model errors. Clim. Dyn. 42, 1189–1202.
20. Jeong, D.I., Sushama, L., Diro, G.T., Khaliq, M.N., Beltrami, H., Caya, D., 2016. Projected changes to high temperature events for Canada based on a regional climate model ensemble. Clim. Dyn. 46, 3163–3180.
21. Nolan, P., O'sullivan, J., Mcgrath, R., 2017. Impacts of climate change on mid-twenty-first century rainfall in Ireland: a high-resolution regional climate model ensemble approach. Int. J. Climatol. 37, 4347–4363.
22. Stratton, R.A., Senior, C.A., Vosper, S.B., Folwell, S.S., Boutle, I.A., Earnshaw, P.D., Kendon, E., Lock, A.P., Malcolm, A., Manners, J., Morcrette, C.J., Short, C., Stirling, A.J., Taylor, C.M., Tucker, S.,Webster, S.,Wilkinson, J.M., 2018. A pan-African convection-permitting regional climate simulation with the met office unified model: CP4-Africa. J. Clim. 31, 3485–3508.
23. Kalognomou, E.-A., Lennard, C., Shongwe, M., Pinto, I., Favre, A., Kent, M., Hewitson, B., Dosio, A., Nikulin, G., Panitz, H.-J., Büchner, M., 2013. A diagnostic evaluation of precipitation in CORDEX models over southern Africa. J. Clim. 26, 9477–9506.
24. Endris, H.S., Omondi, P., Jain, S., Lennard, C., Hewitson, B., Chang'a, L., Awange, J.L., Dosio, A., Ketiem, P., Nikulin, G., Panitz, H.-J., Büchner, M., Stordal, F., Tazalika, L., 2013. Assessment of the performance of CORDEX regional climate models in simulating east African rainfall. J. Clim. 26, 8453–8475.
25. Dosio, A., Panitz, H.-J., Schubert-Frisius, M., Lüthi, D., 2015. Dynamical downscaling of CMIP5 global circulation models over CORDEX-Africa with COSMO-CLM: evaluation over the present climate and analysis of the added value. Clim. Dyn. 44, 2637–2661.
26. Magagula, T.F., van Koppen, B., Sally, H., 2006. Water access and poverty in the Olifants Basin: a spatial Analysis of population distribution, poverty prevalence and trends. 7th Water Net/WARFSA/GWP Annual Symposium, pp. 1–3.
27. McCartney, M.P., Arranz, R., 2007. Evaluation of Historic, Current and Future Water Demand in the Olifants River Catchment. South Africa. Colombo, Sri Lanka. International Water Management

Institute, p. 48p (IWMI Research Report 118). http://www.iwmi.cgiar.org/Publications/IWMI_Research _Reports/PDF/PUB118/RR 18.pdf.

28. McCartney, M.P., Yawson, D.K., Magagula, T.F., Seshoka, J., 2004. Hydrology and Water Resources Development in the Olifants River Catchment, vol. 76. IWMI.
29. SAWB (South African Weather Bureau). 1972. WB35. Climate of South Africa. Part 10. District Rainfall for South Africa and the Annual March of Rainfall over Southern Africa. South African Weather Bureau, Pretoria.
30. Kruger, AC and MP Nxumalo. 2017. Historical rainfall trends in South Africa: 1921–2015. Water SA Vol. 43 (2). http://dx.doi.org/10.4314/wsa.v43i2.12
31. Undén, P., Rontu, L., Jarvinen, H., Lynch, P., Calvo, J., Cats, G., Cuxart, J., Eerola, K., Fortelius, C., Garcia-Moya, J.A., Jones, C., Lenderlink, G., McDonald, A., McGrath, R., Navascues, B., Nielsen, N.W., Odegaard, V., Rodriguez, E., Rummukainen, M., Room, R., Sattler, K., Sass, B.H, Savijarvi, H., Schreur, B.W., Sigg, R., The, H. and Tijm, A. (2002) HIRLAM-5 Scienti_c Documentation, HIRLAM-5 Project, c/o Per Unden SMHI, S-601 76 Norrkoping, Sweden, 146pp.
32. Jones, C., Giorgi, F. and Asrar G. (2011) The Coordinated Regional Downscaling Experiment (CORDEX). An international downscaling link to CMIP5. Clivar Exchanges 16, 34–40.
33. Arora, V. K., Scinocca, J.F., Boer, G.J., Christian, J.R., Denman, K.L., Flato, G.M., Kharin V.V., Lee, W.G. and Merryfield, W.J. (2011) Carbon emission limits required to satisfy future representative concentration pathways of greenhouse gases. Geophysical Research Letters, 38(5), L05805, DOI: 10.1029/2010GL046270.
34. Voldoire, A., Sanchez-Gomez, E., Salas y Mélia, D., Decharme, B., Cassou, C., Sénési, S., Valcke, S., Beau, I., Alias, A., Chevallier, M., Déqué, M., Deshayes, J., Douville, H., Fernandez, E., Madec, G., Maisonnave, E., Moine, M.P., Planton, S., Saint-Martin, D., Szopa, S., Tyteca, S., Alkama, R., Belamari, S., Braun, A., Coquart, L. and Chauvin, F. (2013) The CNRM-CM5.1 global climate model: Description and basic evaluation. Climate Dyn., 40, DOI:10.1007/s00382-011-1259-y, 2091–2121.
35. Rotstayn, L. D., Collier, M.A., Jeffrey, S.J., Kidston, J., Syktus, J.I., and Wong, K.K. (2013) Anthropogenic effects on the subtropical jet in the Southern Hemisphere: aerosols versus long-lived greenhouse gases. Environ. Res. Lett., 8, DOI: 10.1088/17489326/8/1/014030.
36. Hourdin, F., Foujols, M. A., Codron, F., Guemas, V., Dufresne, J. L., Bony, S., Denvil, Sebastien, Guez, L., Lott, F., Ghattas, J., Braconnot, P., Marti, O., Meurdesoif, Y. and Bopp, L. (2013) Impact of the LMDZ atmospheric grid configuration on the climate and sensitivity of the IPSL-CM5A coupled model. Clim. Dyn., 40, DOI: 10.1007/s00382-012-1411-3, 2167–2192.
37. Watanabe, S., Hajima,T., Sudo, K., Nagashima, T., Takemura, T., Okajima, H., Nozawa, T., Kawase, H., Abe, M., Yokohata, T., Ise, T., Sato, H., Kato, E., Takata, K., Emori, S. and Kawamiya, M. (2011) MIROC-ESM 2010: Model description and basic results of CMIP5-20c3m experiments. Geosci. Model Dev., 4, DOI: 10.5194/gmd-4-845-2011, 845–872.Sen, P. K., 1968, "Estimates of the regression coefficient based on Kendall's tau", *Journal of American Statistical Association,* **39**, 379-389.
38. Ilyina, T., Six, K.D., Segschneider, J., Maier-Reimer, E., Li, H., and Nuńez-Riboni, I. (2013) The global ocean biogeochemistry model HAMOCC: Model architecture and performance as component of the MPI-Earth System Model in different CMIP5 experimental realizations, J. Adv. Model. Earth Syst., DOI:10.002/jame.20017.
39. Tjiputra, J. F., Roelandt, C., Bentsen, M., Lawrence, D. M., Lorentzen, T., Schwinger, J., Seland, Ø. and Heinze, C. (2013) Evaluation of the carbon cycle components in the Norwegian Earth System Model (NorESM), Geosci. Model Dev., 6, DOI: 10.5194/gmd-6-301-2013, 301-325.

40. Dunne, J. P., John, J.G., Adcroft, A.J., Griffies, S.M., Hallberg, R.W., Shevliakova, E., Stouffer, R.J., Cooke, W., Dunne, K.A., Harrison, M.J., Krasting, J.P., Malyshev, S.L., Milly, P.C.D., Phillipps, P.J., Sentman, L.T., Samuels, B.L., Spelman, M.J., Winton, M., Wittenbarg, A.T. and Zadeh, N. (2012) GFDL's ESM2 Global Coupled Climate-Carbon Earth System Models. Part I: Physical Formulation and Baseline Simulation Characteristics, Journal of Climate, 25(19), 6646-6665.
41. Kendall, M. G., 1975, “Rank correlation methods”, 4th edition, Charles Griffin, London, U.K.
42. Sen PK. Estimates of the regression coefficient based on Kendall’s tau”, Journal of American Statistical Association. 1968; 39: 379-389.
43. Taylor, K.E., Stouffer, R.J. and Meehl, G.A. (2012) An Overview of CMIP5 and the experimental design. Bull. Amer. Meteor. Soc., 93, 485-498.
44. Kruger, AC and MP Nxumalo. 2016. Surface temperature trends from homogenized time series in South Africa: 1931–2015. International Journal of Climatology. DOI: 10.1002/joc.4851
45. Ziervogel G, New M, Archer ERM, Midgley G, Taylor A, Hamann R, Stuart-Hill S, Myers J and Warburton M (2014). Climate change impacts and adaptation in South Africa. WIREs Clim Change, 5:605–620. doi: 10.1002/wcc.295.

Figure 1

Click here to access/download;Figure;Figure 1.jpg

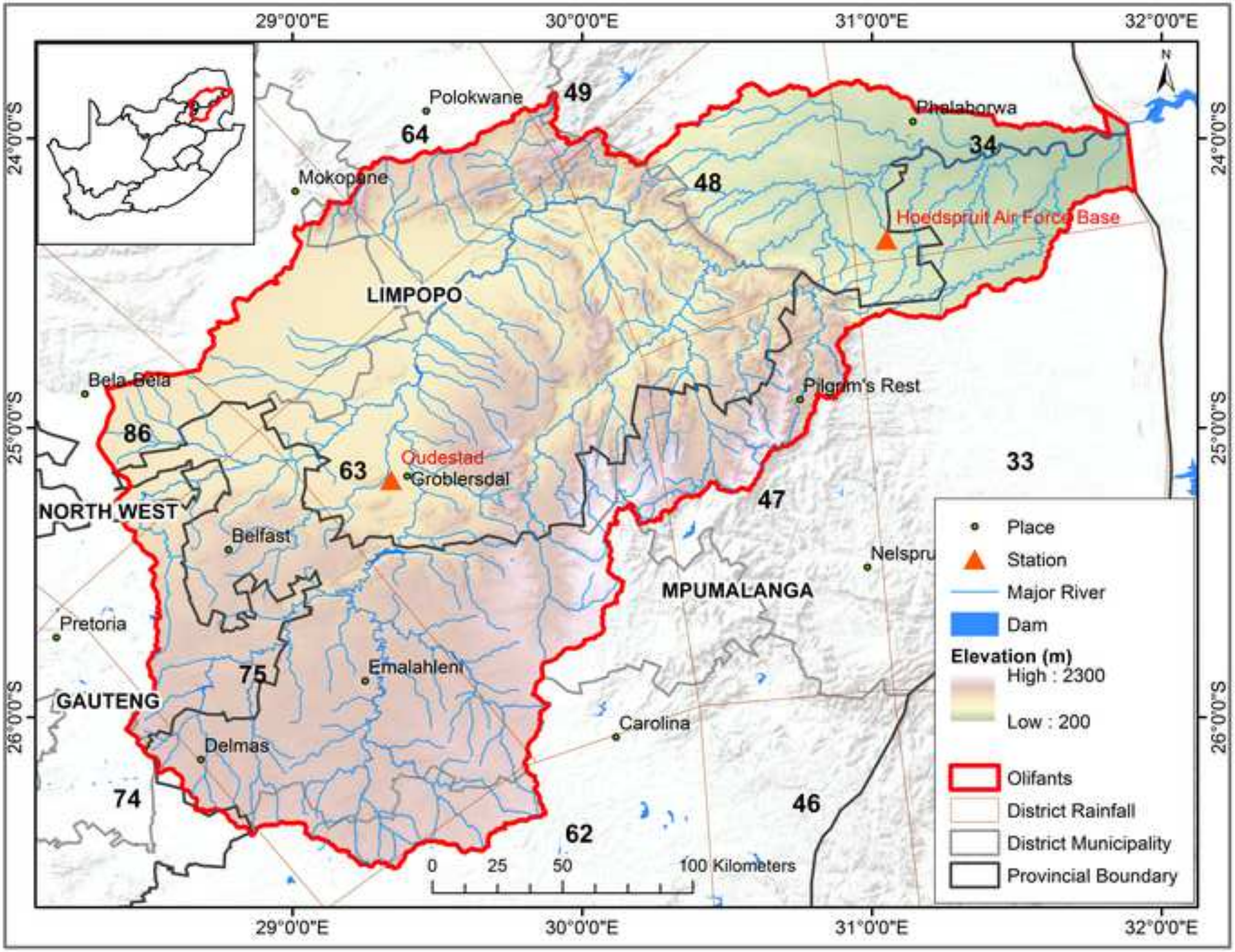

Figure 2

Click here to access/download;Figure;Figure 2.JPG

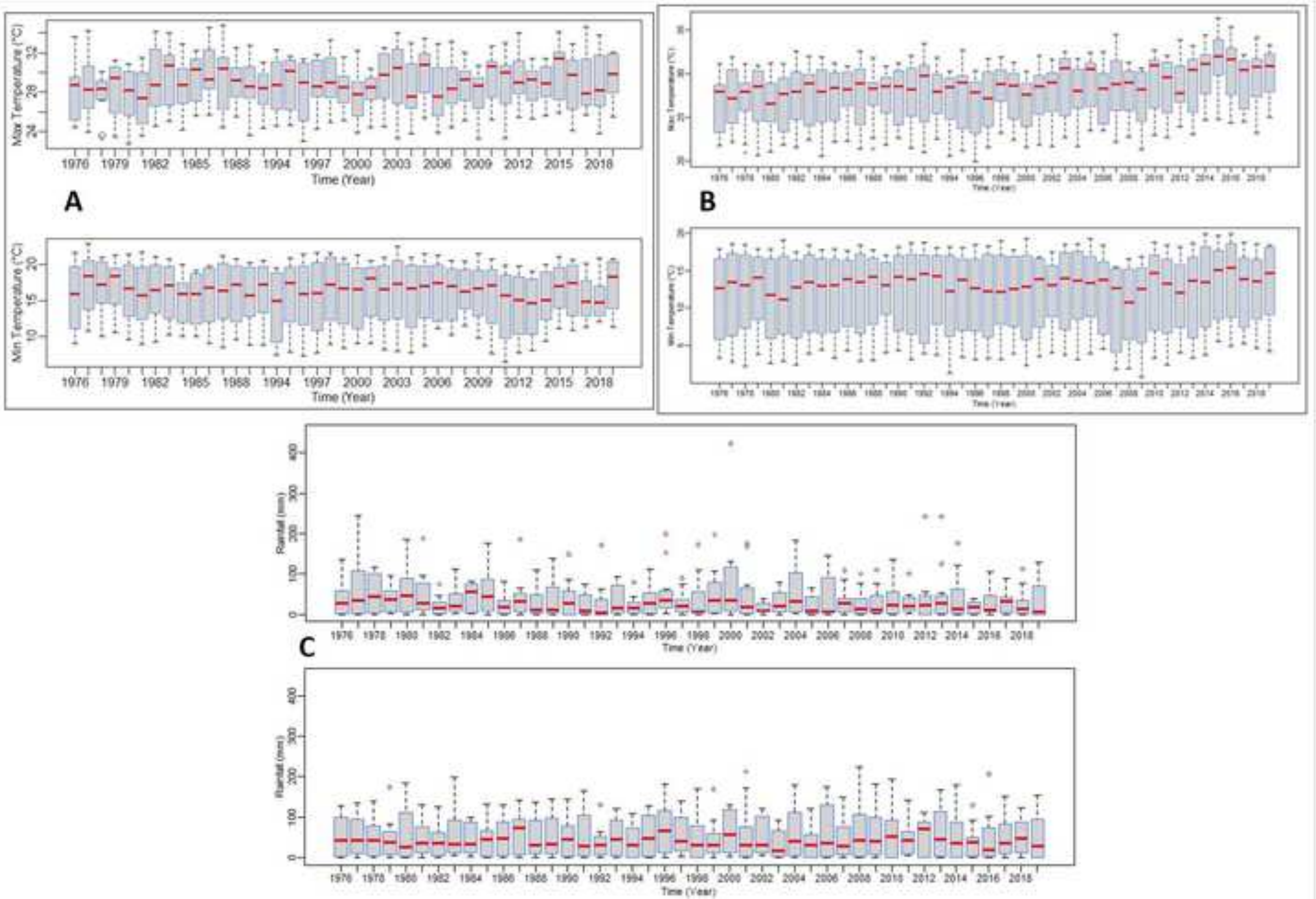

Figure 3

Click here to access/download;Figure;Figure 3.jpg

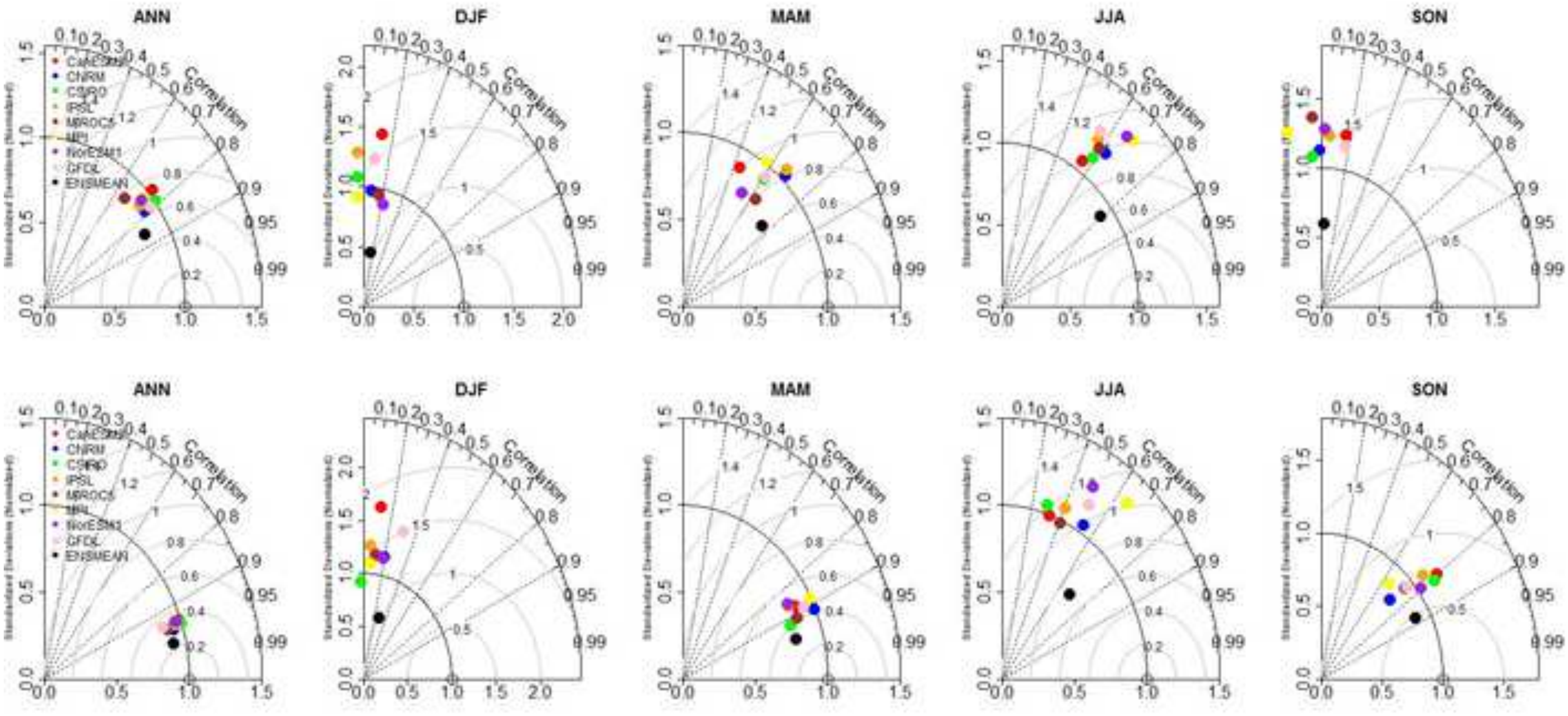

Figure 4

Click here to access/download;Figure;Figure 4.jpg

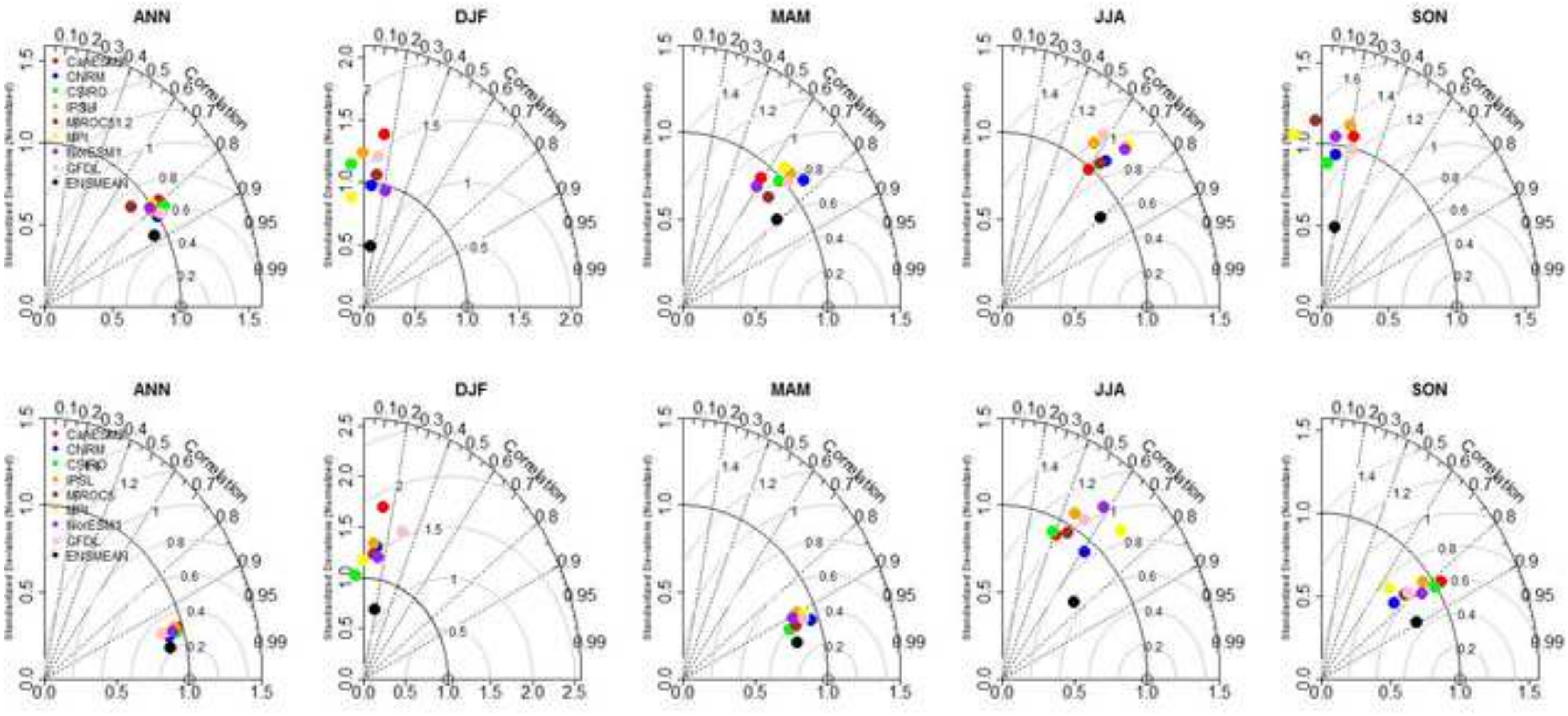

Figure 5

Click here to access/download;Figure;Figure 5.jpg

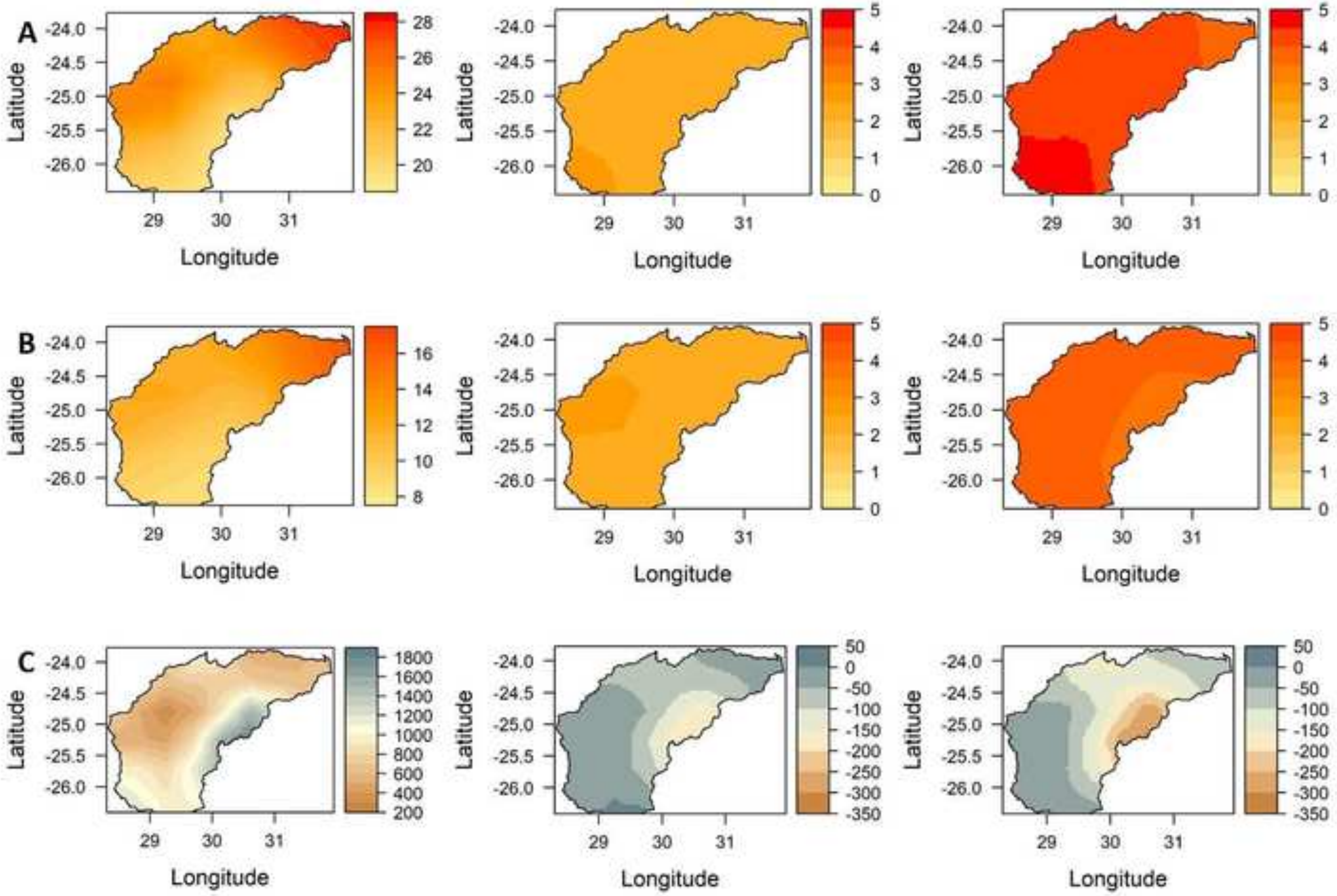

Figure 6 Click here to access/download;Figure;Figure 6.JPG

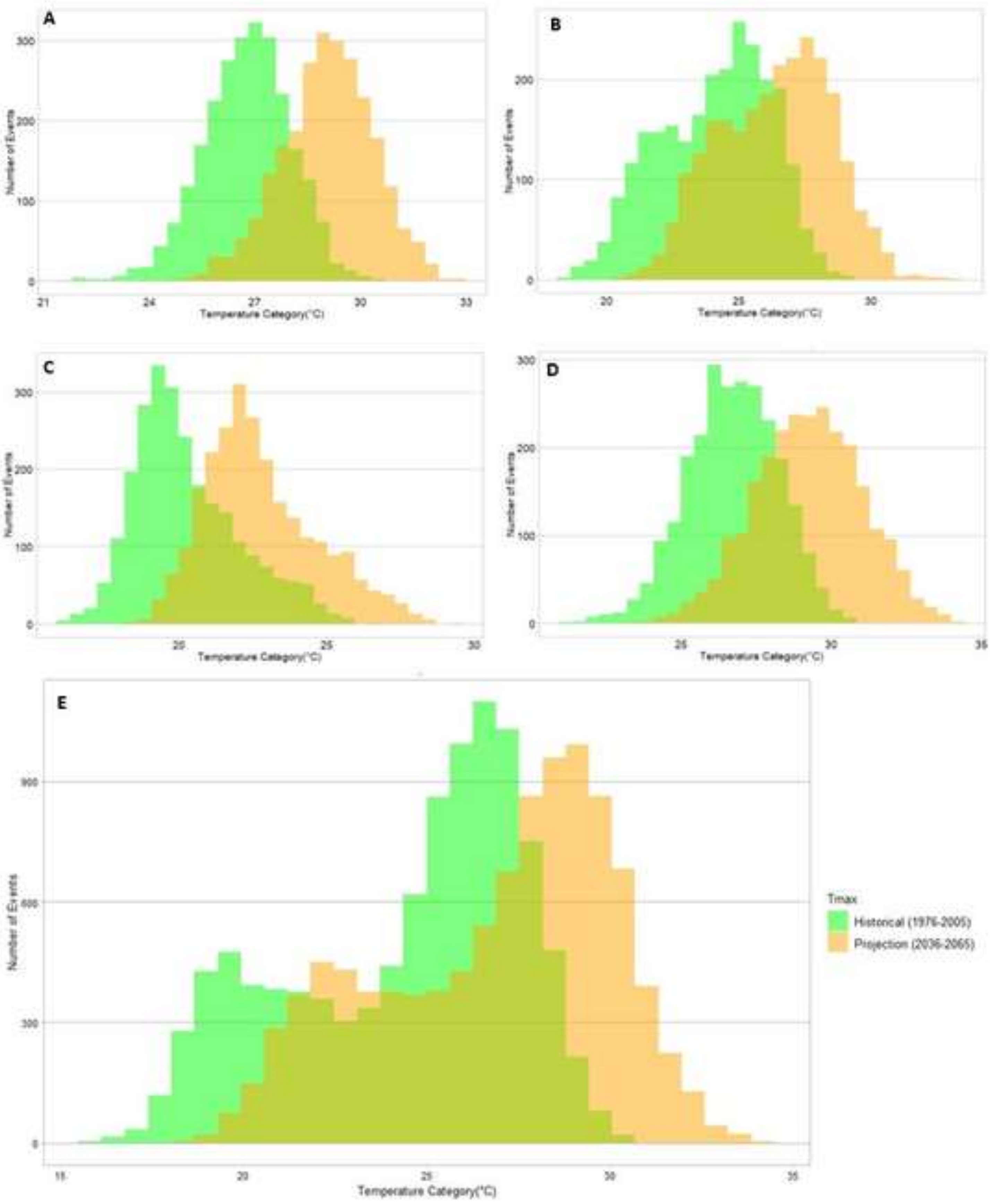

Figure 7

Click here to access/download;Figure;Figure 7.JPG

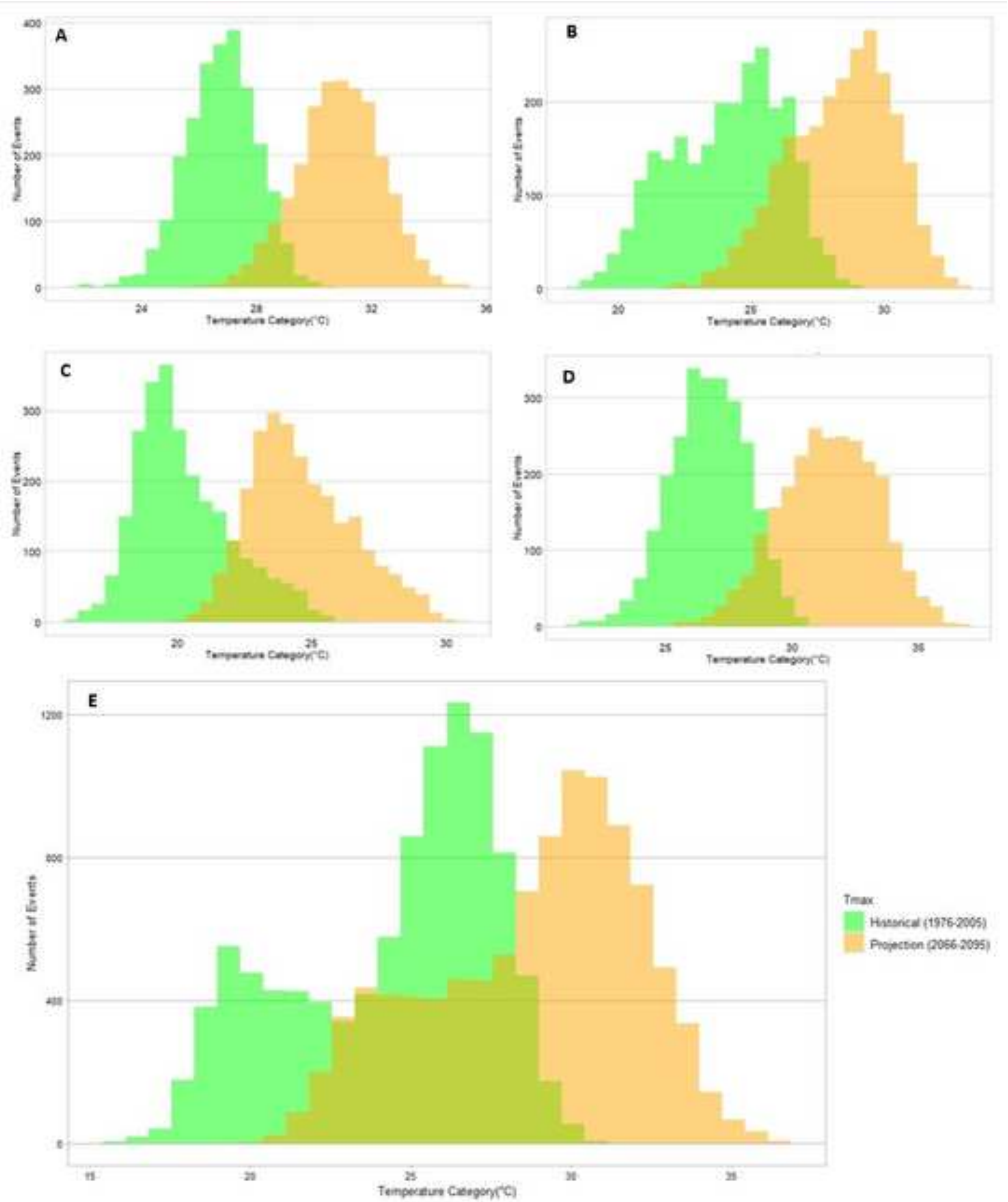

Figure 8

Click here to access/download;Figure;Figure 8.JPG

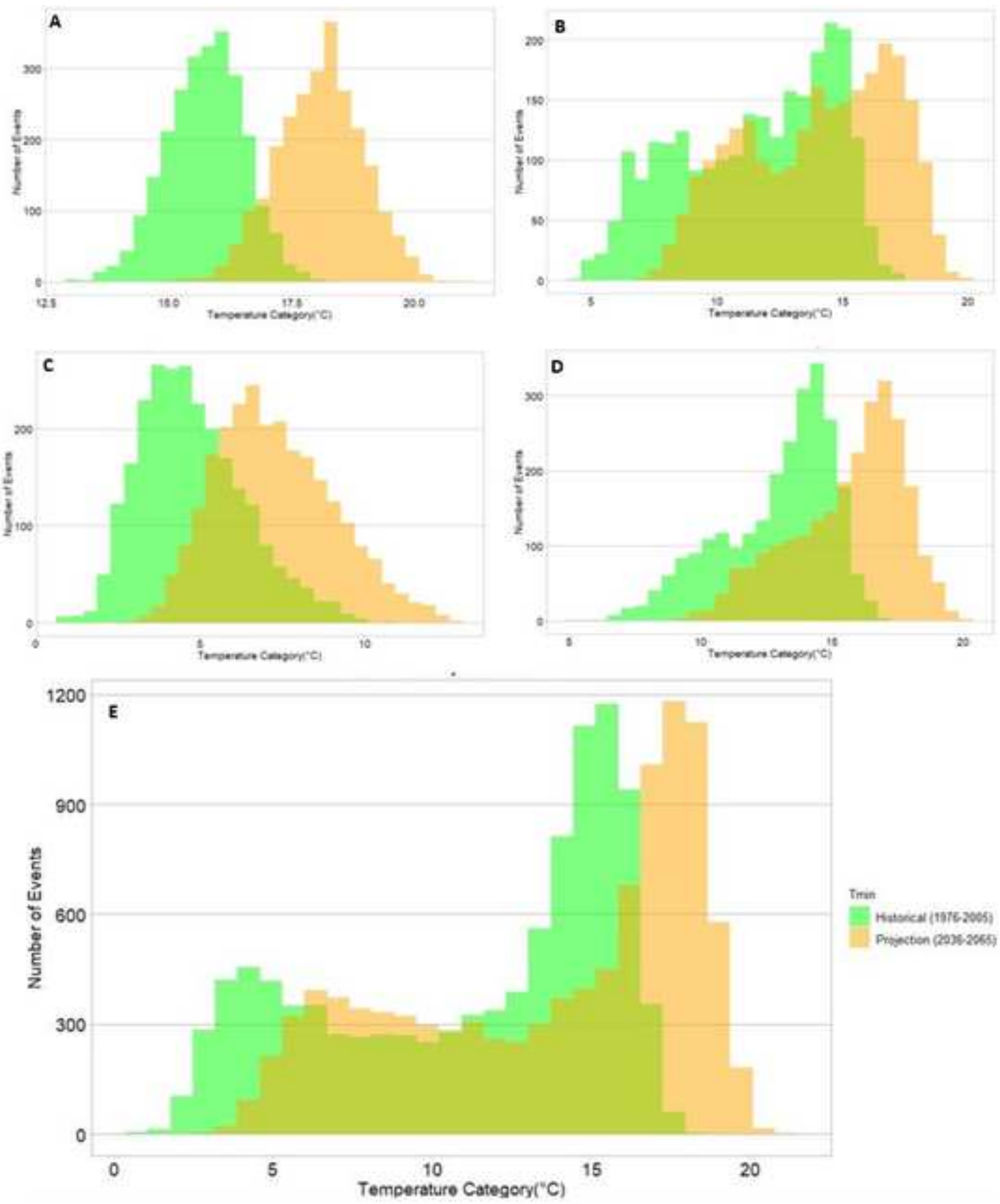

Figure 9

Click here to access/download;Figure;Figure 9.JPG

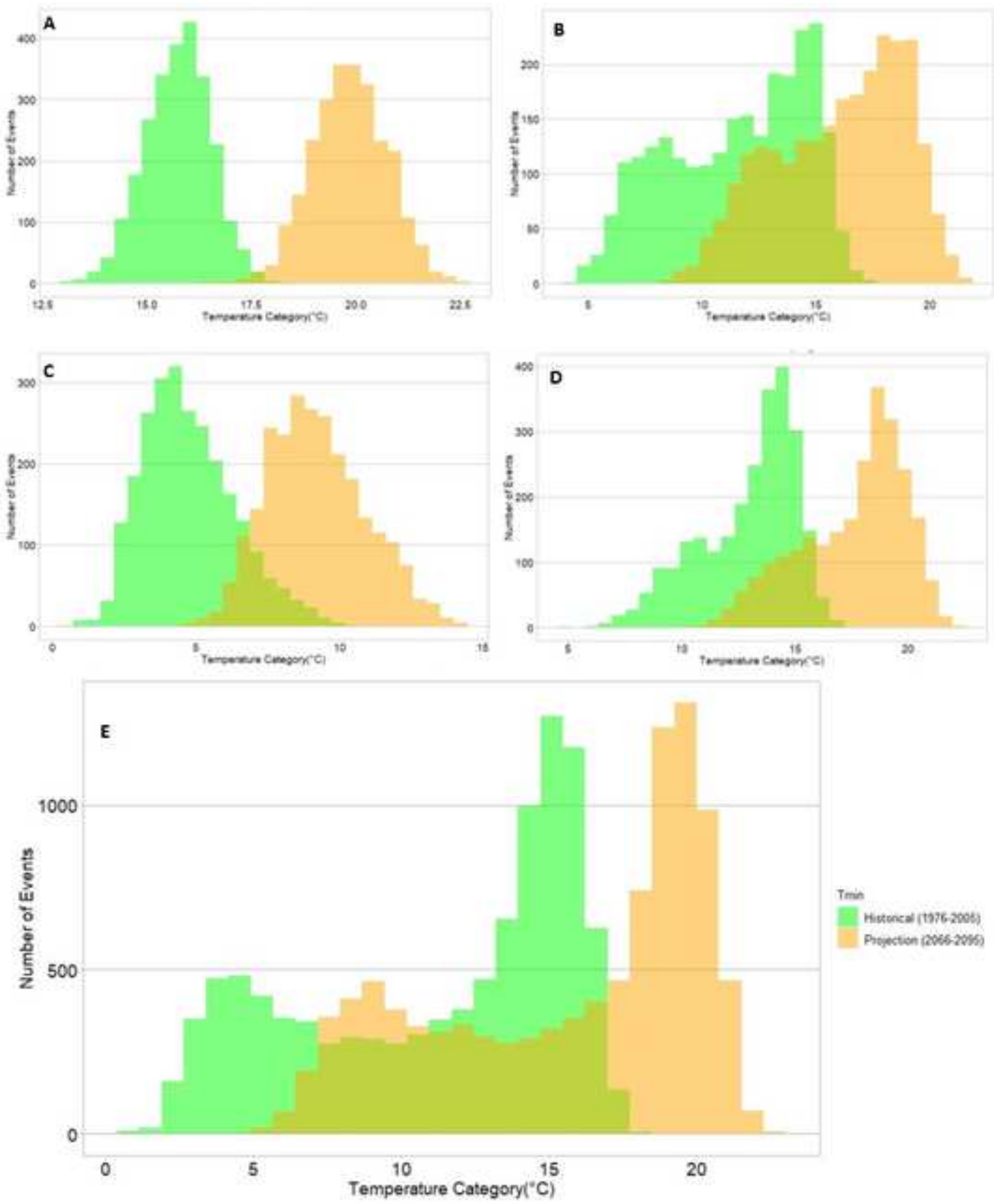

Figure 10

Click here to access/download;Figure;Figure 10.JPG

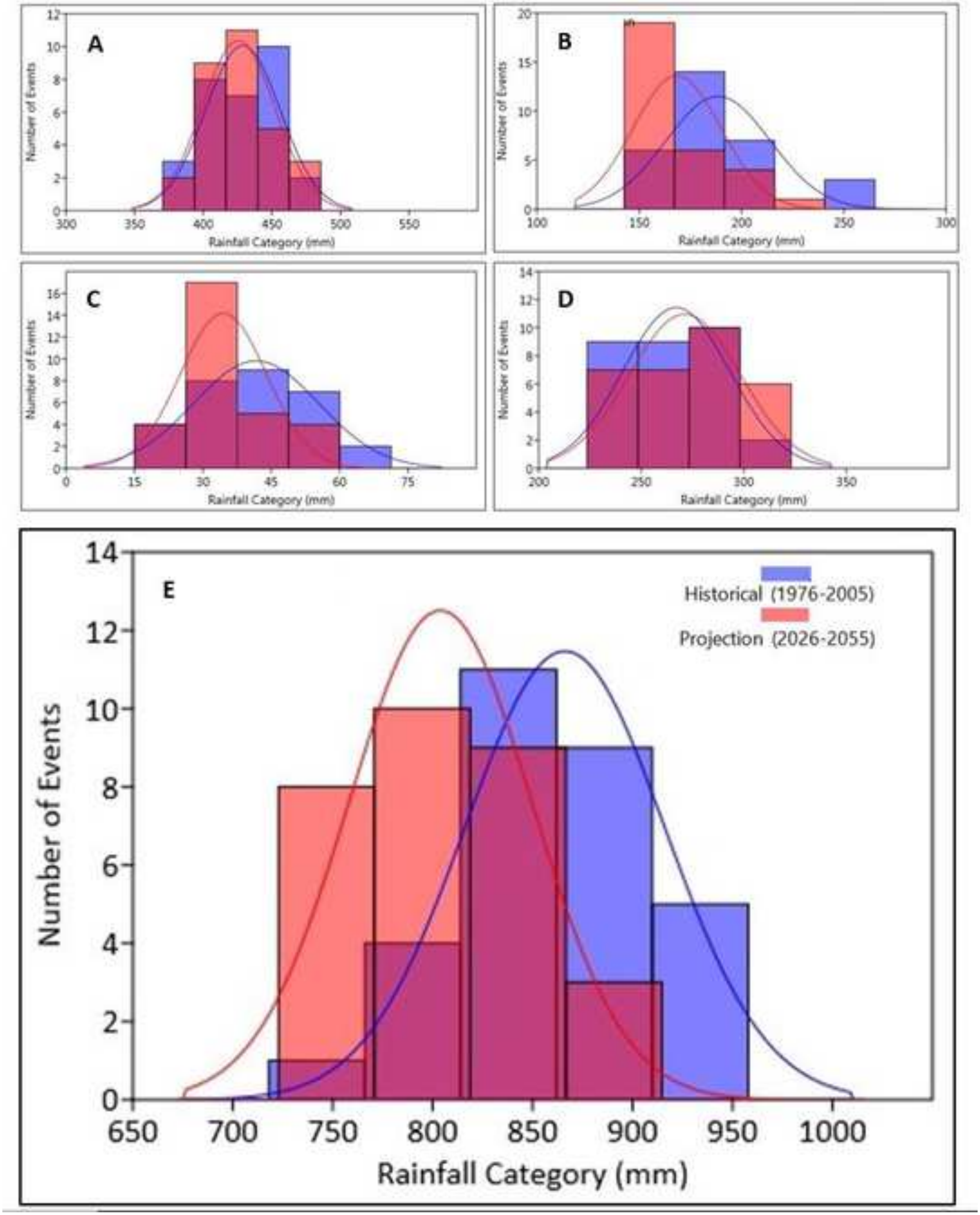

Supplement: Attachment — Submitted filename: PONE-D-21-31259_reviewer-7-38.pdf [file pone.0271974.s002.pdf]
