# Supplementary material for: Observed trends and projections of temperature and precipitation in the Olifants River Catchment in South Africa
Source: PLoS One. 2022 Aug 9;17(8):e0271974. doi: 10.1371/journal.pone.0271974 (PMC9362909; doi:10.1371/journal.pone.0271974)
Supplement: Attachment — Submitted filename: Response to Reviewer 4B.docx [file pone.0271974.s003.docx]

**January 14, 2022**

**ANSWERS TO REVIEWER OF THE MANUSCRIPT**

**“Observed Trends and Projections of Temperature and Precipitation in the Olifants River Catchment in South Africa” by Adeola et al., 2022**

Dear editor,

We would like to thank you for your careful review and constructive comments. We also gratefully acknowledge the anonymous reviewer for his helpful comments which aim at increasing the clarity and readability of the manuscript. The manuscript has been improved according to his recommendations for revision. We think that this version is now well-improved thanks to your useful comments.

Below a detailed point-by-point response to the reviewer’s comments is provided. Changes made in the manuscript are marked using track changes. The revision has been developed in consultation with all co-authors, and each author has given approval to the final version of the manuscript.

Authors: Abiodun Morakinyo Adeola, Andries Kruger, Thabo Elias Makgoale &, Joel Ondego. Botai.

Title:  **Trends and projections of surface temperature and rainfall over the Olifants River catchment**

**General comments**

This is a potentially very useful paper, but the current version and some work with the discussions and additional inputs in the methods and objectives.

The methods and results are interesting, but the presentation of the manuscript is poor, particularly at the discussion level. Based on the discussion section, I think the manuscript is now more written as an internal report, and not a qualitative research paper. This study lacks a discussion section that in quantitative explaining the observed results. The conclusion actually is longer than the discussion not unless I am missing out on the whole context. This is quite a shame because the writing is really well done with logical flow with this information surely very useful in the advancement of the climate prediction agenda based on downscaled models.

**Comment:**

There is no clear objective and specific objectives, research question or hypothesis formulated. Instead, the authors need to come up with a clear research question, rethink the statistics they use on the data based on that question, elaborate in the methods section, and then write a focused discussion.

**Response:**

The comments are well noted and have been implemented in the revised version of the manuscript

**COMMENTS ON MANUSCRIPT;**

Given the title, (Title Trends and projections of surface temperature and rainfall over the Olifants River catchment) and also given the approaches used in the downscaling of data, I think the title is inefficient. I suggest inclusion of the models, i.e., like the following below,

Trends and projections of surface temperature and rainfall over the Olifants River catchment in **(Country ?) CMIP5 model ensemble?**

**Response:**

The comments are well noted and the title has been changed accordingly

**ABSTRACT**

•The abstract is well written covering where and how the research was carried out. The abstract also shows the main findings from the study.

**Introduction**

•	This is well written showing the objectives of the study. et al

**Objective**

The authors have stated the objective as follows,

This study focusses on investigating the trends in historical maximum, minimum temperatures and rainfall, the estimation of plausible future climate changes, as well as the magnitude of future occurrences in the variables to inform adaptation initiatives over the Olifants River Catchment. I think this objective appears very general and lacks specifics and maybe that why the discussion section has been fluffed. I suggest the following,

Include the period in context when capturing the general and specific objectives,

- Also include the ensemble in question in the objectives e.g., dynamically downscaled GCM ensemble using the CMIP5 dataset
- Also include research questions, /hypothesis to be tested.
- the RCP4.5 and RCP8.5 scenarios should also be captured in your objectives. In the absence readers will be lost on what scenarios re been evaluated.

**Response:**

The comments are well noted and have been implemented in the revised version of the manuscript

Table 3,4: Descriptive statistics and trend analysis of maximum, minimum temperature (°C) and rainfall (mm) is better illustrated in a graph.

**Material and methods**

Kindly note that the study area should contain the following information,

Geographical location and description (inform the reader whether area is gazetted, protected or the form of management), climatic information, vegetation types, drainage information, demographic features, land use and resources in an abridged form.

**Response:**

The comments are well noted and the study area section has been revised

Th authors should justify why the picked on the ensemble of eight individual Global Climate Models (GCMs) that participated in the Fifth Phase of Coupled Model Inter-comparison Project (CMIP5). Were there more that participated? Why the eight why not 5? Proper justification required.

**Response:**

The comments are well noted and general update and justification has been done

I have a problem with picking historical data for only two stations out of the 4 which basically means 50 percent data availability. This surely must compromise the data.

**Response:**

The comments are well noted and justification and general rephrasing of the section has been done to provide better clarity

I would like to see the the standardised precipitation index (SPI) classification for extreme weather events (and probability of occurrence) which then can be illustrated through a graph.

**Response:**

The comments are well noted and SPI 2,6 and 12-months have been included in the revised version

Future projections of rainfall, maximum and minimum temperature are presented for the three 30-year periods extending from 2036 – 2065 (near future) and 2066 – 2095 (far future) under the RCP 8.5 scenario. Projected changes are expressed relative to the historical 30-year period of 1976 to 2005. . Based on the above assertions its baffling that the authors limited themselves to baseline scenarios and worst-case scenario (RCP8.5) yet we are living in the intermediate scenario (RCP4.5). Why did the authors omit the RCP 4.5 scenario? We also have RCP 6.5.

**Response:**

The comments are well noted and RCP4.5 has been added in the revised version

**Results**

Results have been well enumerated, however with the absence of aspects captured in the objectives and methods section. It is still inadequate. Most of the figures are not clear and are not easy to comprehend. A simple graphical presentation of rainfall patterns, historical and projected gives a very simple but clearer picture of the peaks, extreme events and also the linear line give a good impression of the trends. This should be adopted for the seasonal, decadal, annual analysis as well.

**Response:**

The comments are well noted and all figures have been improved and well illustrated

**Discussion**

Based on the discussion section, I think the manuscript is now more written as an internal report, and not a qualitative research paper. This study lacks a discussion section that in quantitative explaining the observed results. The conclusion actually is longer than the discussion not unless I am missing out on the whole context. This is quite a shame because the writing is really well done with logical flow with this information surely very useful in the advancement of the climate prediction agenda based on downscaled models. As you enhance your discussion, critically assess the output for the model’s vis a vis extreme rainfall events both for the annual, decadal and seasonal. Also establish whether the models are biased for the short rains over the long rains or vice versa. Compare your results to the east African models which have been christened the “East African climate paradox” as alluded by Rowel et al. 2015, where the observed rainfall trends exhibited a drying anomaly compared to the scenario simulation which projects a wetting anomaly.

In the discussions, I would like to see the following, discussion points on

i Historical rainfall and temperature trends
ii Historical seasonal trends
iii Extreme rainfall events historical in nature
iv Projected rainfall (including seasonality) and temperature trends based on the RCP 4.5 and 8.5
v Projected Extreme rainfall events (forecast on droughts, floods, storms etc)
vi Efficacy of the downscaled models I projecting rainfall but importantly mimicking historical rainfall which is the basis for the projection.

**Response:**

The comments are well noted and the discussions section of the manuscript has been revised

**Conclusion and Recommendation**

I have reserved my comments for conclusion and recommendation because according to this paper, the discussion section is missing. it is impossible to make conclusions.

**Response:**

The manuscript has generally been updated. Thank you again for the positive feedback and thank you for your consideration. We look forward to receiving a good review of this paper.
