# Supplementary material for: Observed trends and projections of temperature and precipitation in the Olifants River Catchment in South Africa
Source: PLoS One. 2022 Aug 9;17(8):e0271974. doi: 10.1371/journal.pone.0271974 (PMC9362909; doi:10.1371/journal.pone.0271974)
Supplement: Attachment — Submitted filename: Responses_to_Reviewer 4_R2.docx [file pone.0271974.s004.docx]

**April 08, 2022**

**ANSWERS TO REVIEWER OF THE MANUSCRIPT**

**“Observed Trends and Projections of Temperature and Precipitation in the Olifants River Catchment in South Africa” by Adeola et al., 2022**

Dear editor,

We would like to thank you for your careful review and constructive comments. We also gratefully acknowledge the anonymous reviewer for his helpful comments which aim at increasing the clarity and readability of the manuscript. The manuscript has been improved according to his recommendations for revision. We think that this version is now well-improved thanks to your useful comments.

Below a detailed point-by-point response to the reviewer’s comments is provided. Changes made in the manuscript are marked using track changes. The revision has been developed in consultation with all co-authors, and each author has given approval to the final version of the manuscript.

Authors: Abiodun Morakinyo Adeola, Andries Kruger, Thabo Elias Makgoale &, Joel Ondego. Botai.

Reviewers' comments:

Reviewer #4: The manuscript has been improved a lot as per the suggestion of the reviewers. It is now ready for acceptance (if other reviewers have similar views).

***Response:***

*We thank the Reviewer to this valuable comment. We have implemented the comment and we are glad to return a much improved discussion section in the revised manuscript.*
